# Wireless Sensor Network Congestion Control Based on Standard Particle Swarm Optimization and Single Neuron PID

**DOI:** 10.3390/s18041265

**Published:** 2018-04-19

**Authors:** Xiaoping Yang, Xueying Chen, Riting Xia, Zhihong Qian

**Affiliations:** School of Communication Engineering, Jilin University, Changchun 130025, China; yxp@jlu.edu.cn (X.Y.); xychen16@mails.jlu.edu.cn (X.C.); xiart15@mails.jlu.edu.cn (R.X.)

**Keywords:** wireless sensor networks, congestion control, PID algorithm, neuron algorithm, standard particle swarm optimization, NS2

## Abstract

Aiming at the problem of network congestion caused by the large number of data transmissions in wireless routing nodes of wireless sensor network (WSN), this paper puts forward an algorithm based on standard particle swarm–neural PID congestion control (PNPID). Firstly, PID control theory was applied to the queue management of wireless sensor nodes. Then, the self-learning and self-organizing ability of neurons was used to achieve online adjustment of weights to adjust the proportion, integral and differential parameters of the PID controller. Finally, the standard particle swarm optimization to neural PID (NPID) algorithm of initial values of proportion, integral and differential parameters and neuron learning rates were used for online optimization. This paper describes experiments and simulations which show that the PNPID algorithm effectively stabilized queue length near the expected value. At the same time, network performance, such as throughput and packet loss rate, was greatly improved, which alleviated network congestion and improved network QoS.

## 1. Introduction

In recent years, with the rapid development of wireless sensor network (WSN) technology, researchers have proposed many protocols for wireless sensor networks. For example, the problems of life span and energy consumption in a network are key areas of research focus. In order to prolong the life of a network, Habib Mostafaei et al. [1] used the imperialist competitive algorithm (ICA) for selecting sensor nodes to engage in barrier coverage monitoring operations called ICABC. The main objective of this work was to improve the network lifetime in a deployed network. Prolong-SEP (P-SEP) [2] prolongs the stable period of Fog-supported sensor networks by maintaining balanced energy consumption. 

In addition, wireless sensor network congestion has become increasingly significant. In WSNs, congestion is caused by the following factors: packet collision, node buffer overflow, transmission channel contention, transmission rate, many-to-one data transmission scheme, and dynamic time variation transmission channel. Indeed, congestion has a significant impact on Quality of Service (QoS) parameters such as the packet delivery ratio (PDR), end-to-end delay, and energy consumption in wireless nodes [3]. Therefore, effective congestion avoidance is an important performance indicator of WSNs, and the congestion control technology of WSNs has become a hot research topic in recent years.

Li et al. [4] proposed a new congestion control algorithm with e-approximation and weighted fairness to solve the problem of data compression and weighted fairness in existing WSN congestion algorithms. Sun [5] proposed a new congestion control method that made use of the combination of buffer queue length and its variation rate to estimate the degree of congestion. It divided the node states and adopted various bandwidth allocation strategies according to different states, which ensured the reliable transmission of emergent information. Active queue management (AQM) schemes was implemented in the routers of communication networks to react to incipient congestion before the queue overflowed [6]. Li et al. [7] proposed a congestion avoidance strategy based on the RED (Random Early Detection) algorithm, which is more commonly used in router queue management to manage cache space queue length. It was introduced into the wireless sensor network and adopted the congestion degree threshold as the basis of congestion regulation. By comparing the different experimental results under different experimental parameters, we can achieve better WSN transmission performance under the appropriate parameter values.

The current congestion control strategy is based on technology that uses automatic control theory to design a network controller capable of fulfilling the control requirements of a WSN data stream. Classical PI (Proportion Integration) control technology is introduced into a WSN, and the data cache queue length in the network node is taken as the controlled object [7,8,9]. The PI controller is designed to achieve active queue management. An active queue management method combining PI control techniques with a quantum particle swarm optimization algorithm was proposed in [10]. Firstly, an improved PI controller model was defined. Then, a quantum particle swarm optimization algorithm was used to optimize the parameters in the PI controller model. The experimental results showed that the proposed method could effectively achieve WSN congestion control with a low data packet loss rate and large network throughput. A quantum particle swarm congestion control algorithm considering fairness was also proposed. Firstly, quantum particle swarm optimization (QPSO) was introduced into the PID (Proportion Integration Differentiation) queue management algorithm to adapt to the complex environment of WSN. Then, according to the network congestion conditions and the packet transmission distance, the queuing probability between nodes was adjusted with different transmission distances from Sink in order to improve the network load fairness [11].

In this paper, by combining the characteristics of each algorithm in [8,9,10,11], we designed a congestion control method based on a standard particle swarm–neural PID controller, which improved the speediness, convergence and accuracy of the PID controller, and network performance in terms of packet loss rate and delay in the WSN. The PID control parameters were optimized using the neuron control technique and the standard particle swarm optimization algorithm.

## 2. Related Work

The work related to this paper mainly includes three parts: the design of a PID controller with a WSN as the controlled object, single neuron control techniques, and standard particle swarm optimization.

### 2.1. The WSN Node Queue Model of PID Control

**Figure 1:**
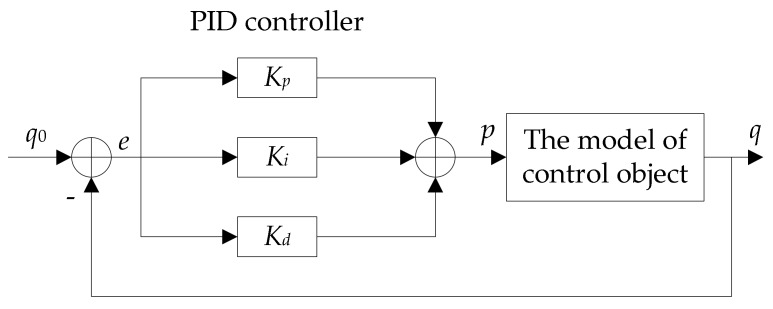
PID queue congestion control model.

In recent years, it has been proposed that PI and PID controller technology be applied to the WSN node cache queue [8,9,10,11]. The purpose of this is to effectively control the stability of WSN node cache queue length and improve network performance. The PID control model is shown in Figure 1. The physical meaning of each parameter in Figure 1 is shown in Table 1.

After the necessary discrete processing of the analog PID control algorithm, continuous time t is represented by a series of sampling time points kT, the sum is substituted for the integral, and the incremental is substituted for the differentiation. The discrete PID expression is obtained as:

(1) $$p\left(k\right)=K_{p}e\left(k\right)+K_{i}T\sum_{j=0}^{K}e\left(j\right)+K_{d}\{\frac{\left[e\left(k\right)-e\left(k-1\right)\right]}{T}\}$$

As incremental form:

(2) $$\Delta p\left(k\right)=K_{p}\{(1+\frac{T}{T_{i}}+\frac{T_{d}}{T})e\left(k\right)-\left(1+\frac{2T_{d}}{T}\right)e\left(k-1\right)+\frac{T_{d}}{T}e\left(k-2\right)\}$$

Among them, $T_{i}=K_{p}/K_{i}$, $T_{d}=K_{d}/K_{p}$, T are given for the sampling time.

Although the application of PI and PID queue management algorithms to WSN nodes has shown some ease of network congestion performance, the parameters of traditional PI and PID algorithms are fixed, so they cannot adapt to the dynamic network environment.

### 2.2. Single Neuron Control Technique

The adaptive control of single neurons can be achieved by adjusting the weighting coefficient so the adaptive and self-organizing functions are realized. The adjustment of the weight coefficient is calculated according to a certain learning rule. Not only is this structure simple, but it also can adapt to environmental changes. The core of single neuron control technology is a learning algorithm. According to different learning signals, neuron learning algorithms can be divided into three categories: unsupervised Hebb learning algorithm, supervised Hebb learning algorithm, and supervised Delta learning algorithm. Using a single neuron with supervised Hebb learning to adjust the weight of the PID controller can improve the dynamic characteristics of the system [12]. In [13], the single neuron control technique was adopted to dynamically adjust the parameters of a PID controller and was applied to the internet network router to control the length of the buffer queue, so as to improve the stability and throughput of the queue. This method has been widely used in wired networks.

In a WSN, when an event is detected, network traffic increases. This in turn increases the flow of data packets and congestion. In this paper, error was calculated by finding the difference between the actual and the desired packet drop. To reduce the error obtained from the fuzzy logic based congestion control algorithm, a Back Propagation Network (BPN) based congestion control algorithm was proposed [14]. Ali Ahmadi el al. [15] reviewed the efficient routing algorithms for preserving k-coverage in a sensor network and then proposed an effective technique for preserving k-coverage and the reliability of data with logical fault tolerance. In order to detect the location of WSN node faults and diagnose the cause of the failure, a fault diagnosis method based on the current model of wireless communication modules has been proposed in [16]. This method uses BP neural networks to dynamically adjust the fault diagnosis parameters of the wireless communication module in different states. In [17], the sink node was the bottleneck of the WSN. Because of the characteristics of sensor networks, congestion control strategies in traditional wired networks are no longer applicable. This paper proposed a queue controller of neural networks based on gray prediction. The method first uses the self-learning ability of RBF (Radial Basis Function) neural networks to solve the problem of online tuning of algorithm parameters when the network changes in real time. Then, the gray GM (Gray Model) (1, 1) predictor is used to effectively solve the influence of large time delays on the network performance. Finally, the simulation results show the effectiveness of the proposed algorithm.

### 2.3. Parameter Design of Standard Particle Swarm Optimization Algorithm

Inspired by simulating social behavior results, Kennedy and Eberhart proposed the Particle Swarm Optimization (PSO) algorithm. The global random search algorithm is based on swarm intelligence and was derived by simulating the migration and clustering behaviors of birds during feeding [18].

In [19], the conventional servo system was analyzed according to a model of motor shaft rotation, and the optimization and adjustment of the PID parameters of the stabilized platform was carried out using the improved PSO algorithm. Through verification, simulation and experimentation with hardware platforms, we have shown the stability of platforms based on spatial analysis models. Improving the particle swarm algorithm to optimize PID controllers can lead a stable platform to have higher accuracy and better robustness, more effectively isolating external vibration and interference. In a WSN [20], it is usually necessary to process the data measured by the network nodes to judge whether the WSN is running reliably. Aiming to solve this problem using traditional algorithms involves complex calculations and high energy consumption. A new method based on particle swarm optimization and Gaussian distributions has been proposed by the author Yu, C.B to diagnose faults in WSN nodes. Wireless sensor network nodes need to forward large amounts of data, which can lead to network congestion, resulting in high packet loss rates and low network throughput. The author Li, X.L. proposed a new congestion control method based on an active PI model and the improved quantum-behaved particle swarm optimization in [10]. Simulations showed that the proposed congestion control method could effectively achieve WSN congestion control. Therefore, it is an effective congestion control method for WSN. Compared with other methods, it has a shorter average queue length, a larger network throughput, and strong feasibility.

The SPSO (Standard Particle Swarm Optimization) algorithm is based on the particle swarm algorithm and introduces inertia weight *w*, which is used to balance the global search ability and the local search ability of the SPSO algorithm. Therefore, inertia weight selection is very important. A large number of experiments have shown that when *w* is large, it is beneficial to global search ability, but when *w* is small, it is beneficial to local search ability.

## 3. Queue Management Methods for Standard Particle Swarm–Single Neuron PID Wireless Sensor Network Nodes

In this paper, a congestion control algorithm for the queue management of WSN nodes based on standard particle swarm neuron PID is proposed, called PNPID (standard particle swarm–single neuron PID). This method is described below.

Because traditional PID controllers have a high degree of reliance on the accuracy of the object model, traditional PID control cannot achieve good results for complex and variable systems. The choice of parameters has a large influence on the performance of a controller and when its robustness is not good, this causes PID controller limitations. In order to solve the shortcomings of fixed parameters, a neural network control technique was applied to the parameter tuning of the PID algorithm. A single neuron has a high self-adaptive and self-learning ability. The weights of single neurons are, respectively, matched with the PID coefficients of proportion, integral and differentiation. Thus, the PID controller can adjust the coefficient adaptively online to increase the system’s adaptive capacity and robustness.

With the existence of neurons, there will be side effects, such as slow convergence. Therefore, we propose to use the SPSO algorithm to solve this problem by optimizing neuron learning rates.

### 3.1. WSN Node Queue PNPID Control Model

**Figure 2:**
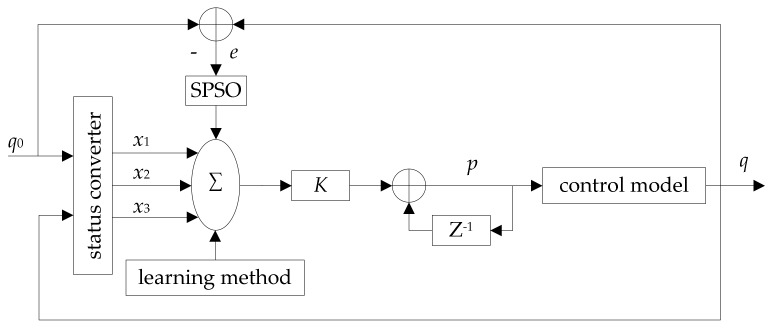
PNPID algorithm control principle diagram.

PNPID uses the node actual queue length as the controlled object (congestion indicator), which can better perceive the dynamic changes of the wireless sensor network. The control model is shown in Figure 2.

The physical meanings of q, $q_{0}$, p, e are the same as in Figure 1. $x_{1}$, $x_{2}$ and $x_{3}$ are the three inputs of a single neuron and the calculated drop probability p is the output of the control system. Z represents Z-transformation and K is the gain of a single neuron, ensuring that K is greater than zero.

### 3.2. Single Neuron PID Control System (NPID)

In Figure 2, the inputs of PNPID are expressed as follow:

(3) $$\begin{array}{l} x_{1}\left(k\right)=q\left(k\right)-q_{0}=e\left(k\right)\\ x_{2}\left(k\right)=e\left(k\right)-e\left(k-1\right)=ec\left(k\right)\\ x_{3}\left(k\right)=e\left(k\right)-2e\left(k-1\right)+e\left(k-2\right) \end{array}$$

where $x_{1}\left(k\right)$ is the error between actual queue length and expectation, $x_{2}\left(k\right)$ is the first order difference of the error and $x_{3}\left(k\right)$ is the second order difference of the error.

The input–output relationship of the neuron control system is:

(4) $$u\left(k\right)=u\left(k-1\right)+K\sum_{i=1}^{3}w_{i}\left(k\right)x_{i}\left(k\right)$$

where $w_{i}\left(k\right)$ is the weight of $x_{i}\left(k\right)$, and u(k) is the output of a single neuron.

### 3.3. Single Neuron Control Learning Method

Learning rules are processes that constantly modify neuron weight through some algorithm that studies the surrounding environment to adapt to that environment. In the neuron learning process, weight $w_{i}\left(k\right)$ is in direct proportion to the progressive signal $V_{i}\left(k\right)$, and slowly decays at the same time. Its learning rule can be represented as follow:

(5) $$w_{i}\left(k+1\right)=w_{i}\left(k\right)+\eta_{i}V_{i}\left(k\right)$$

where $\eta_{i}$ is the learning rate coefficient, which should be greater than zero, and Vi(k) is the progressive signal.

According to need, we use the supervised Hebb learning algorithm [21], which has already been mentioned in Section 2.2, to tune the weighting coefficients $w_{i}\left(k\right)$, and its expression is as follow:

(6) $$V_{i}\left(k\right)=z\left(k\right)u\left(k\right)x_{i}\left(k\right)$$

where z(k) is the error signal output and z(k)=e(k). Therefore, the final expression of the learning algorithm is defined as follows:

(7) $$w_{i}\left(k+1\right)=w_{i}\left(k\right)+\eta_{i}z\left(k\right)u\left(k\right)x_{i}\left(k\right)$$

A single neuron controller [22] achieves adaptive adjustment by adjusting $w_{i}\left(k\right)$, which is the weight of the input variable $x_{i}\left(k\right)$. In order to achieve the convergence and robustness of the algorithm [23], we use the standardized learning algorithms for processing. The expression can be written as follows:

(8) $$p\left(k\right)=p\left(k-1\right)+K\sum_{i=1}^{3}w_{i}{}^{\prime }\left(k\right)w_{3}\left(k\right)$$

where $w_{i}{}^{\prime }\left(k\right)$ is:

(9) $$w_{i}{}^{\prime }\left(k\right)=\frac{w_{i}\left(k\right)}{\sum_{j=1}^{3}\left|w_{j}\left(k\right)\right|}$$

$\eta_{1}$, $\eta_{2}$ and $\eta_{3}$ are coefficients of different learning rates. In order to get a good control effect in the simulation, we can set the learning rate according to the situation.

### 3.4. SPSO Algorithm is Added to the NPID Control System

The specific steps are as follows:

1. The position and velocity of the particles in the particle group are uniformly initialized. Assuming that the population size of a particle swarm is *N*, each particle represents a solution in the *D*-dimensional search space, where the position and velocity of the particle i in the solution space can be expressed as:
  $$X_{i}=\left(x_{i1},x_{i2},\ldots ,x_{iD}\right),V_{i}=\left(v_{i1},v_{i2},\ldots ,v_{iD}\right)$$
  Among them, *N* represents the population size of the particle swam, which relates to the number of iterations. The value of *N* does not depend on *D*. After a large number of experiments and reference to relevant empirical values, the value of *N* = 30 is selected. *D* represents the dimension. The PNPID algorithm has six initialization parameters $\left(K_{p0},K_{i0},K_{d0},\eta_{1},\eta_{2},\eta_{3}\right)$, so *D* = 6. During the search process, the particle’s flying speed has a range $\left[-V_{\max},V_{\max}\right]$. Generally, the selection of $V_{\max}$ will also have a large influence on optimization performance. If the particle’s velocity is too fast, it is easy to leap into the optimal solution. If it is too small, it will easily stagnate and fall into a local optimum. However, the introduction of inertial weights in the SPSO algorithm can remove the influence on $V_{\max}$. The purpose of this is to prevent the occurrence of search disorder.
2. Calculate the fitness of each particle. This paper selected for the fitness of $f=\frac{1}{\int_{0}^{\infty }t\left|e\left(t\right)\right|dt}$;
  Among them, e(t) is the deviation of the given value and output value of the system. In this paper, e(t) is the difference between the expected queue length and actual queue length.
3. The best position of the particle is $p_{i}=\left(p_{i1},p_{i2},\ldots ,p_{iD}\right)$, and the best position of the particles in the population, that is, the global best position is $G=\left(p_{g1},p_{g2},\ldots ,p_{gD}\right)$. The best position of the particle is determined by Formula (10):
  (10) $$P_{i}\left(t\right)=\left\{ \begin{array}{l} X_{i}\left(t\right)\;if\;f\left(X_{i}\left(t\right)\right)<f\left(P_{i}\left(t-1\right)\right)\\ P_{i}\left(t-1\right)\;if\;f\left(X_{i}\left(t\right)\right)\geq f\left(P_{i}\left(t-1\right)\right) \end{array}\right.$$
  The subscript g in the global best position $G=\left(p_{g1},p_{g2},\ldots ,p_{gD}\right)$ of the population is determined by Formula (11):
  (11) $$g=\arg\underset{1\leq i\leq D}{\min}\{f\left(P_{i}\left(t\right)\right)\}\;g\in \{1,2,\ldots ,D\}$$
4. The evolution of the velocity and position of the particle. In the SPSO model, the velocity and position of particles in each dimension are updated as follows:
  (12) $$V_{id}\left(t+1\right)=wV_{id}\left(t\right)+c_{1}r1_{id}\left(t\right)\left(P_{id}\left(t\right)-X_{id}\left(t\right)\right)+c_{2}r2_{id}\left(t\right)\left(P_{gd}\left(t\right)-X_{id}\left(t\right)\right)$$
  (13) $$X_{id}\left(t+1\right)=X_{id}\left(t\right)+V_{id}\left(t+1\right)$$
  where $V_{id}$ and $X_{id}$ represent the velocity component and the position component of the *d*th dimension of the *i*th particle, respectively. The best position component of the *i*th particle is $P_{id}$. $P_{gd}$ is the historical best position component of the group. w is the inertia weight, and the inertia weight adopts a decreasing strategy, which determines the inheritance of the particle to the current velocity; r1 and r2 are uniformly distributed and independent random numbers in the interval (0, 1), and are called random factors; $c_{1}$ is the individual cognitive acceleration factor, which indicates the memory of the best position of the person who has experienced the history; and $c_{2}$ is the population cognitive acceleration coefficient, which indicates the memory ability of the particle to the historical best position experienced by the whole population. The existence of learning factors gives the particles the ability to self-summarize and learn from the best individuals in the group. Through the complementarity and coordination of the two abilities, the particles are close to the global optimal position or the local optimal position. In this paper, the learning factors are $c_{1}=0.95+0.1\times rand,c_{2}=c_{1}$ [24].
5. To determine the end condition, the fitness of the objective function is good enough or evolved to a predetermined algebra, otherwise the return step (2) continues.

### 3.5. Pnpid Algorithm Description

**Figure 3:**
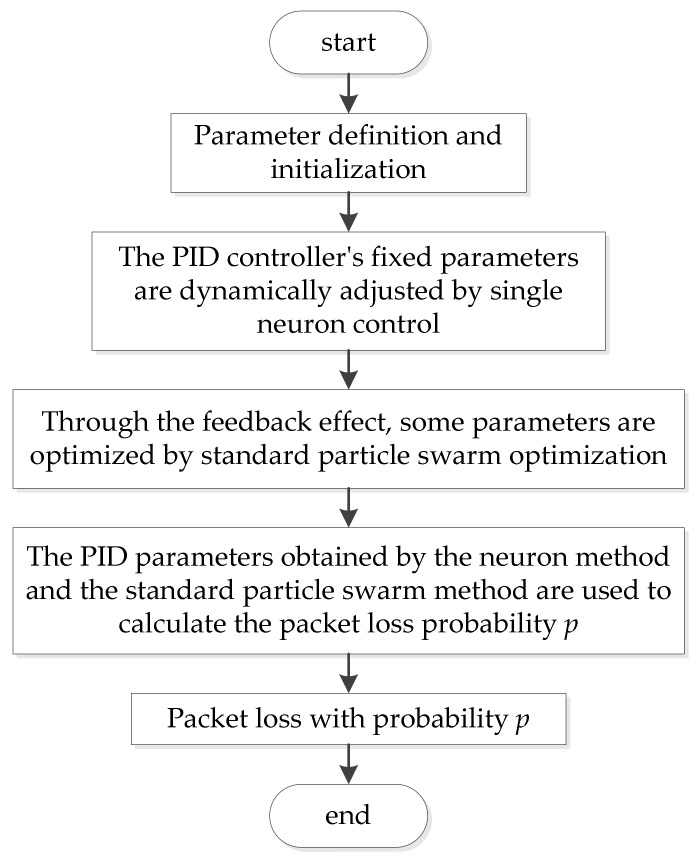
Flow chart of PNPID algorithm.

1. The PID congestion control algorithm based on PID control technology combined with an active queue management algorithm is embedded into the WSN environment;
2. The parameters of the PNPID algorithm are initialized according to the condition of the wireless sensor network and the desired queue length, sampling frequency and other related parameters are set;
3. The parameters $K_{p}$, $K_{i}$ and $K_{d}$ of the PID queue congestion control model are adjusted online through a single neuron control technique. According to Formula (4) in the text, the single neuron controller achieves adaptive adjustment by adjusting the weight $w_{i}\left(k\right)$ of the input variable $x_{i}\left(k\right)$. $w_{1}\left(k\right)$, $w_{2}\left(k\right)$ and $w_{3}\left(k\right)$ correspond to $K_{p}$, $K_{i}$ and $K_{d}$, respectively. More detailed procedures are given in Section 3.3.
4. Using a modified particle swarm optimization algorithm, the learning rate in the neuron PID algorithm is optimized. According to Formulas (10)–(13), the weights used to adjust the single neurons online are corrected in real time to prevent the local optimization problem of a single neuron algorithm. Algorithm performance becomes better. More detailed procedures are given in Section 3.4. The PNPID algorithm is obtained.
5. According to Formula (8), the discard probability p for active packet loss is calculated. In this way, we can advance the packet loss ahead of the queue buffer overflow, so as to avoid congestion. The flow chart of specific implementations is shown in Figure 3:

## 4. Simulations

In this paper, a WSN congestion simulation experiment was designed. The PNPID algorithm proposed in this paper was compared with a PI, PID and neuron PID (NPID) algorithm. By using the queue length, packet loss rate, delay and throughput, it was verified that the PNPID algorithm could effectively alleviate network congestion and improve network QoS in the WSN node queue.

### 4.1. Simulation Environment and Parameter Settings

**Figure 4:**
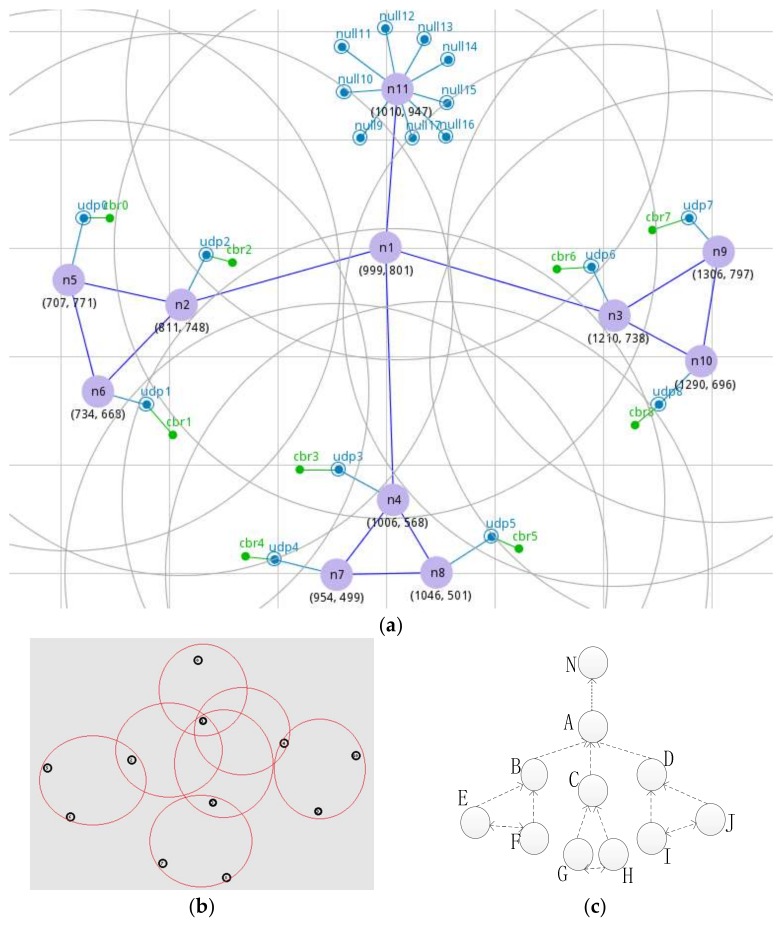
WSN topology: (**a**) a manual set of network topology in NSG2; (**b**) a topological graph of NAM in NS2; (**c**) a painted topology.

The experiment simulation platform was on the Ubuntu 10.04 operating system. The simulation software used NS2 and the version was NS-2.35. Because the tree topology used in this paper was extremely similar to the structure of the wireless sensor network and can reflect the typical topology of a WSN, the topology is displayed in Figure 4. Figure 4a is the network topology diagram manually set in NSG2, which is a software that can generate TCL scripts automatically. Figure 4b is the topology map generated by NAM in NS2, the black circle represents the node, and the red circle represents the communication range. The sensor nodes in the same red circle can transmit data. In Figure 4c, a topology is drawn, where nodes B, C, and D send data to node A, and a bottleneck node is formed at node A. Node N is the only sink node, and the other sensor nodes are both a sensing node and a routing node. The definition of each parameter in the topology diagram of WSN is given in Table 2. Table 3 defines the parameters of each algorithm. It was not necessary to set the initial value of the PID algorithm parameters as these were adjusted online by the neuron weights.

### 4.2. Simulation Results and Performance Analysis

#### 4.2.1. PI, PID, NPID, and PNPID Algorithms Actual Queue Length Comparison

**Figure 5:**
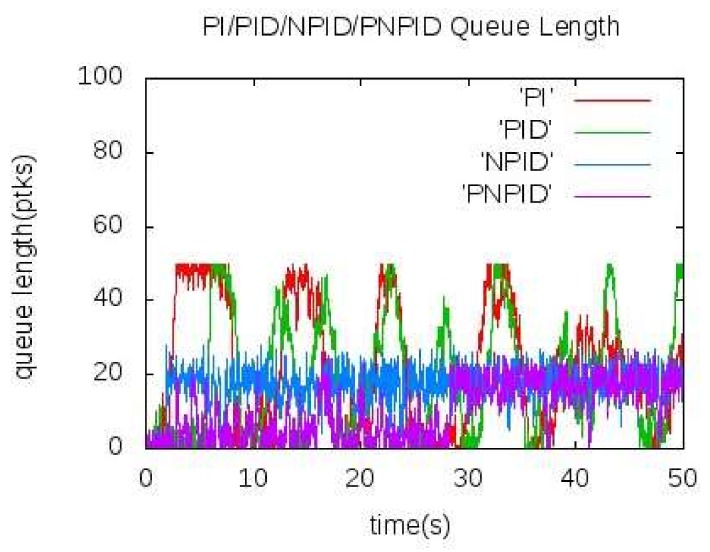
Queue length of node A at 60 kb/s.

**Figure 6:**
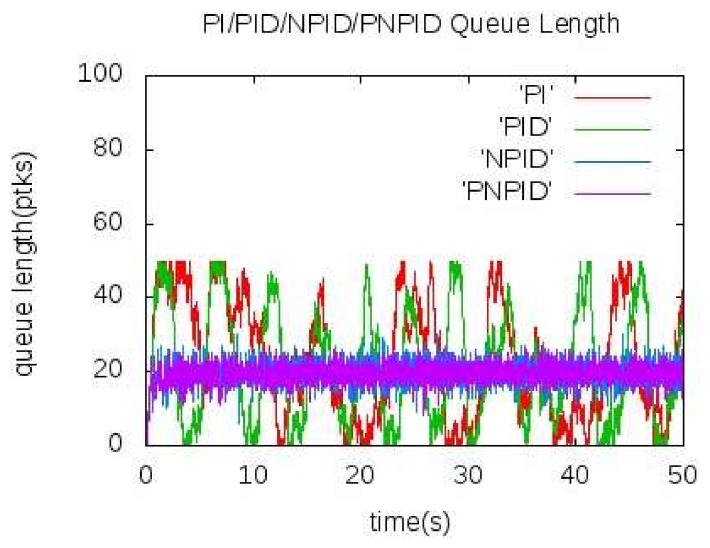
Queue length of node A at 100 kb/s.

Figure 5 and Figure 6 show the actual queue length of the bottleneck node A when the source node sends data to the sink node at 60 kb/s and 100 kb/s, respectively. It can be seen from the figure that the queue length of node A cannot converge well near the expected value in the traditional PI and PID algorithm and show a large concussion. In Figure 5, the queue length of the NPID algorithm kept near the expected value. In the PNPID algorithm proposed in this paper, the queue length was shorter than the expected value before 30 s and the queue length was stable near the expected value after the 30 s. As shown in Figure 6, the queue length was maintained near the expected value and the amplitude of the shock was less than the NPID algorithm.

**Figure 7:**
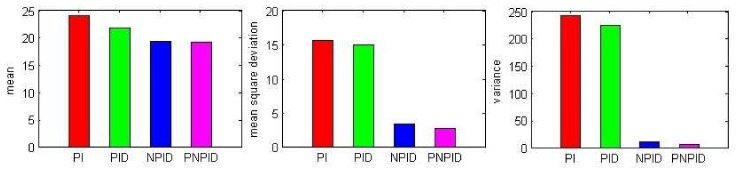
Mean, mean square deviation and variance of the actual queue length at a rate of 100 kb/s.

The PNPID algorithm of actual queue length remained stable. Figure 7 shows the mean and mean square error of the actual queue length when the source node sent data to the sink node at a rate of 100 kb/s. It can be seen from the figure that the PNPID algorithm queue length was the most stable.

The traditional PI and PID algorithm parameters were fixed and could not adapt to the changing environment of the wireless sensor network very well. The PI algorithm lacked the adjustment of the differential link, making the PI algorithm slow to converge to the expected queue value. 

#### 4.2.2. PI, PID, NPID, and PNPID Algorithms Packet Loss Rate and Packet Delivery Ratio Comparison

In this paper, the packet loss rate of node A was calculated as d = (total number of packets lost by node A/number of packets sent by source node). The calculation formula of the entire network packet loss rate was d = (the number of packets lost by all nodes/the number of packets sent by the source node).

**Figure 8:**
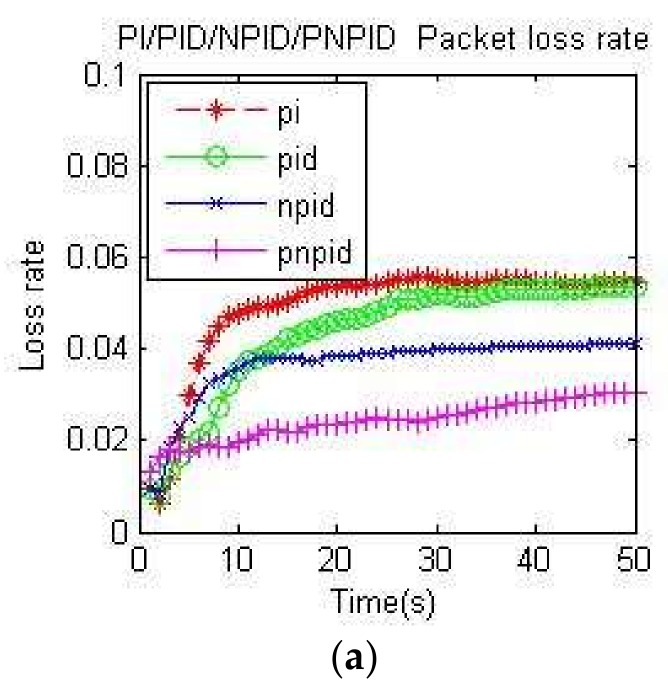

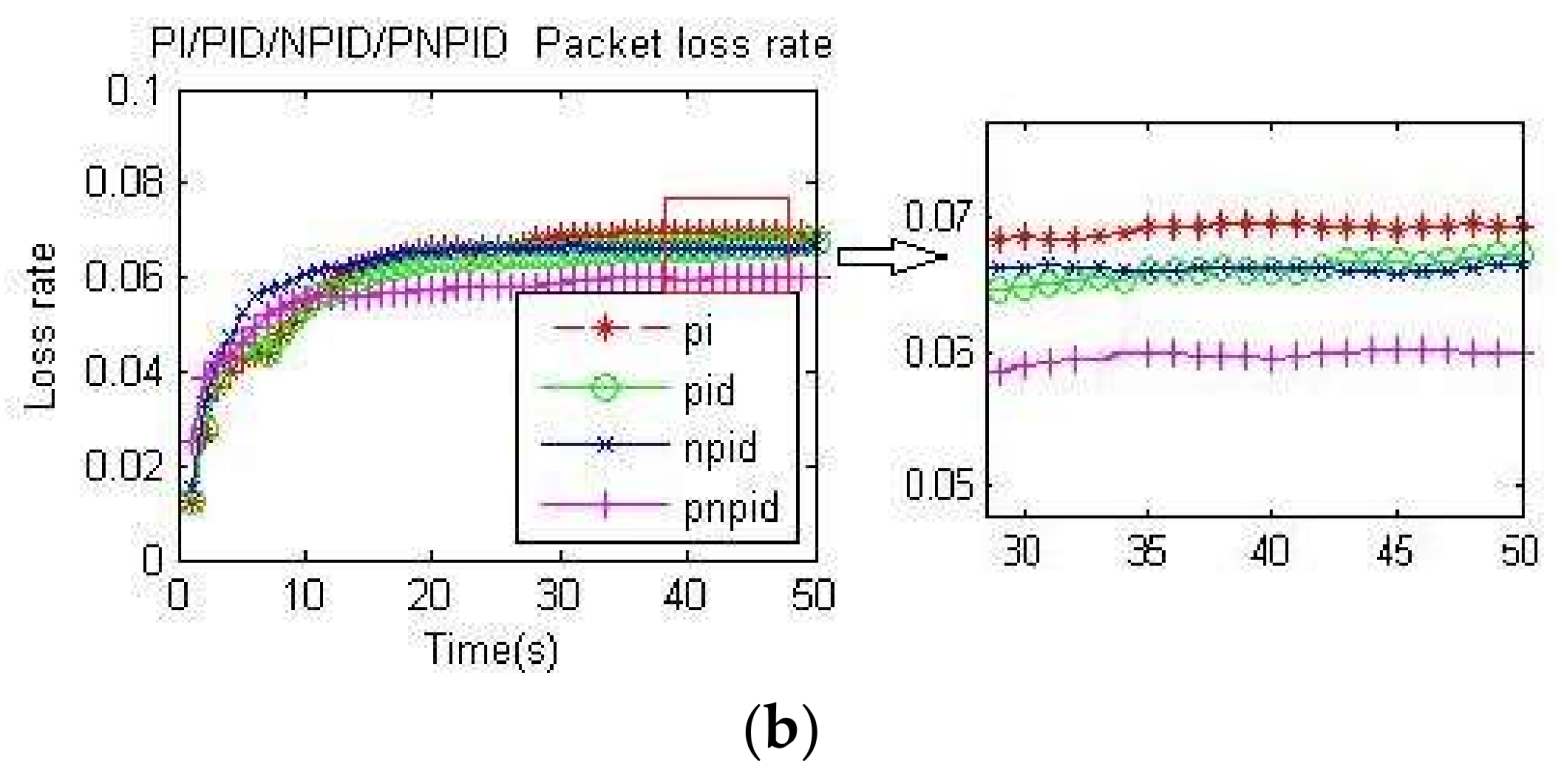
Packet loss rate at node A: (**a**) packet loss rate of the four algorithms at node A at 60 kb/s; (**b**) packet loss rate of the four algorithms at node A at 100 kb/s.

**Figure 9:**
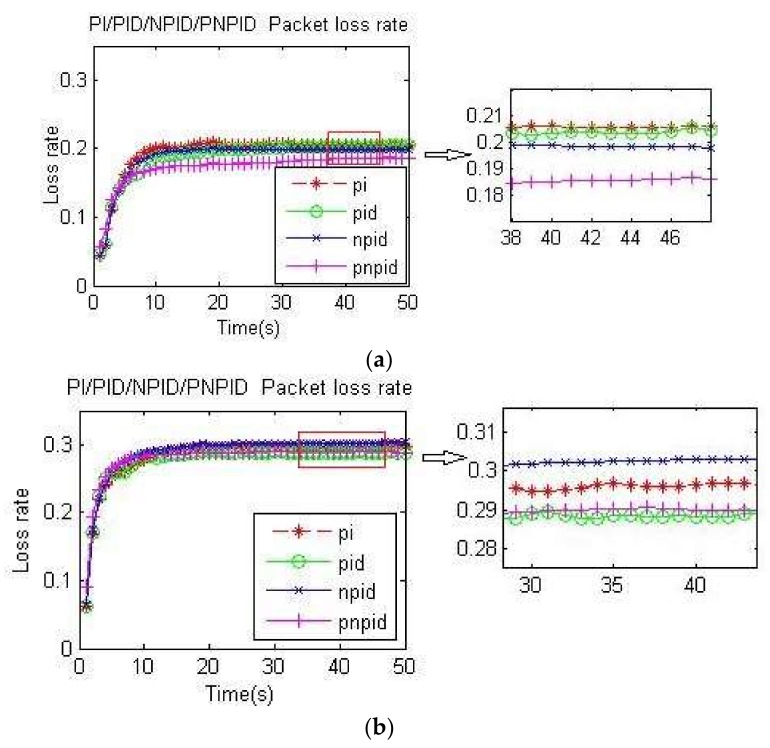
Packet loss rate of the whole network: (**a**) packet loss rate of the whole network at 60 kb/s; (**b**) packet loss rate of the whole network at 100 kb/s.

Figure 8a,b and Figure 9a,b, respectively, measure the packet loss rate of the four algorithms at node A and the whole network at two different transmission rates. It can be seen that the PNPID algorithm proposed in this paper was almost the lowest at every moment.

Because the PI algorithm and PID algorithm oscillate around the desired queue length and have full queues, the packet loss rate was high. Although the neuron algorithm had good self-adaptability and the packet loss rate was lower than traditional algorithms, it had slow convergence and a long learning time. In addition to speeding up the adjustment time and adjustment accuracy by the differential link, the PNPID algorithm also added a standard particle swarm optimization algorithm to adjust and optimize the initial value of the PID controller and the learning rate of the neuron online, so that the queue length was stable near the expected value. Because the PNPID stabilized the queue length, the queue cache margin of the node was larger, thus reducing the packet loss rate.

**Figure 10:**
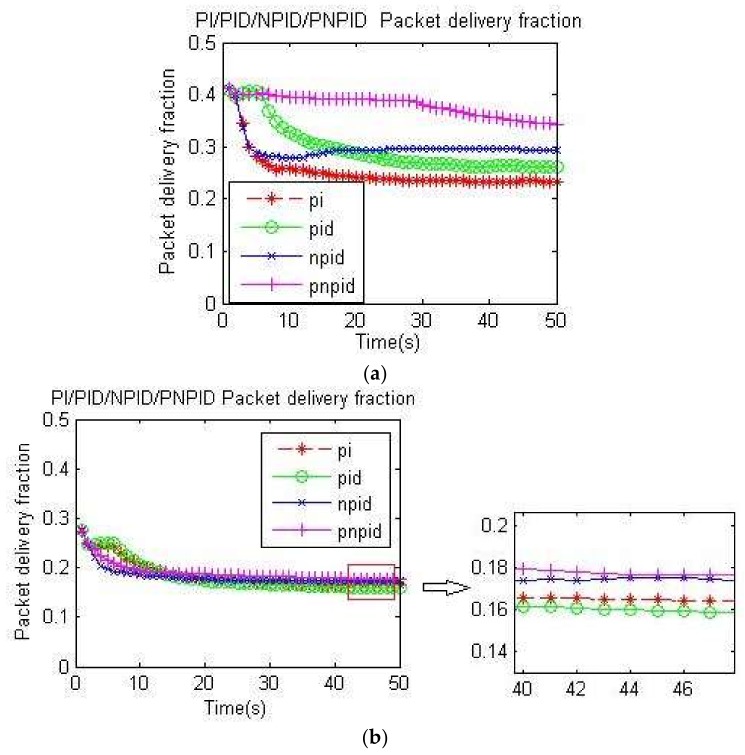
Packet delivery rate: (**a**) packet delivery rate at 60 kb/s; (**b**) packet delivery rate at 100 kb/s.

The packet delivery fraction was calculated as f = (number of sink nodes received/number of packets sent by the source node). Figure 10a,b shows the packet delivery rate for the four algorithms at different rates, where the packet delivery rate of the PNPID algorithm was higher than that of several other algorithms.

The packet loss rate of the PNPID algorithm was reduced, indicating that the number of lost packets had lowered so that the arrival rate of packets increased.

#### 4.2.3. Throughput and Delay Experimental Contrast

In this paper, the throughput calculation formula was t = (all nodes in a certain period of time to receive the total amount of data/time).

**Figure 11:**
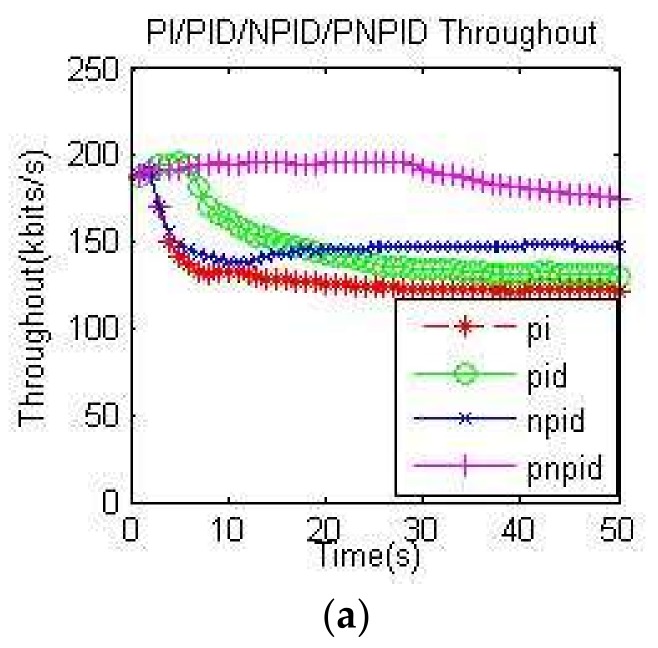

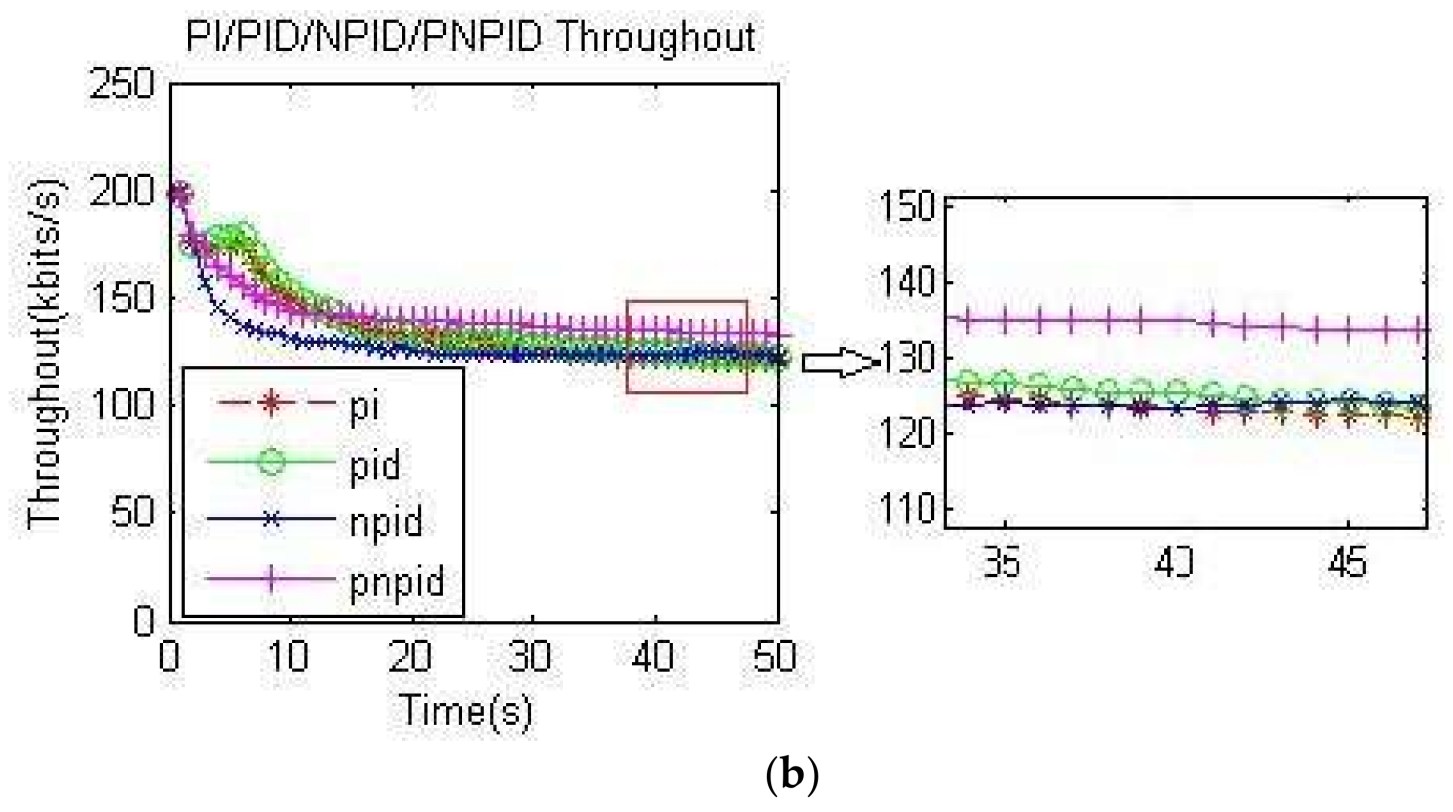
Throughput: (**a**) throughput at 60 kb/s; (**b**) throughput at 100 kb/s.

In Figure 11a,b, which shows the different transmission rates of the entire network using a throughput comparison diagram, we can see that the throughput of all algorithms basically declined. The main reason for the decrease was the high packet transmission rate, which led to channel collisions. The packet loss rate increased. However, the throughput of the PNPID algorithm proposed in this paper was higher than the other algorithms, regardless of the transmission rate. 

The parameters of the traditional PI and PID algorithms were fixed, so the length of the queue was unstable and there were many packet losses. As a result, the throughput was low. Because the length of the queue controlled by the PNPID algorithm was near the expected value, it was more conducive to the successful transmission of the packet, thereby increasing the throughput of the network transmission.

The transmission delay of network formula is:

$$\bar{D}=\frac{\sum_{i=1}^{N}D\left(i\right)}{N}=\frac{\sum_{i=1}^{N}\left[RT\left(i\right)-ST\left(i\right)\right]}{N}$$

where D(i) is the transmission delay of the *i*th data packet, RT(i) is the reception time of the *i*th packet, and ST(i) is the transmission time of the *i*th packet.

**Figure 12:**
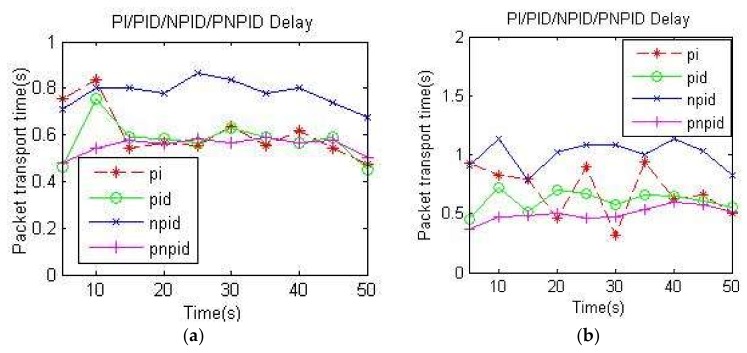
Delays: (**a**) delays at node A at 60 kb/s; (**b**) delays at node A at 100 kb/s.

Figure 12 shows the delay diagrams for the four algorithms. It can be seen from the figures that the delay of the NPID algorithm was obviously higher than that of other algorithms at two different transmission rates, and that the delay of the PI algorithm and PID algorithm had a large oscillating range. These cannot be tolerated for systems with strict delay requirements, such as voice systems. The proposed PNPID algorithm had the smallest delay most of the time, which improved its delay performance index.

The convergence speed of the neuron algorithm was slow, and the average queue length of the NPID algorithm was more than the average queue length of the PNPID algorithm, which increased the queue time and delay. The standard particle swarm algorithm added to the PNPID algorithm improved the convergence speed of the neuron algorithm, reducing the average queue length and delay. Therefore, the PNPID algorithm showed an advantage in terms of delay performance.

### 4.3. Summary of the Experiment

Because traditional PI and PID algorithm parameters are fixed, they cannot be well adapted to a WSN environment. The control process of a single neuron PID algorithm is to optimize the weight of a neural network constantly so that the output error of the system decreases. However, the convergence rate of the neuron controller is slow and precision is low. The PNPID algorithm proposed in this paper was based on a neural algorithm and an improved PID algorithm. The SPSO algorithm was used to optimize the learning rate of the neural network and the initial value of the PID parameters. Both the convergence speed and accuracy of the neuron PID algorithm was improved. Therefore, the queue length of PNPID showed superior performance.

On the one hand, the neuron algorithm had good dynamic characteristics, strong adaptability, good anti-interference and strong robustness. On the other hand, the SPSO algorithm improved the convergence speed of the neuron algorithm. Therefore, the PNPID algorithm greatly reduced the packet loss rate of the bottleneck node and the packet loss rate of the whole network, alleviated congestion, improved QoS performance of the whole network, and reduced the delay of packet transmission.

## 5. Conclusions

WSN congestion can reduce the success rate of data transmission, reduce transmission quality of service, and data retransmission can further lead to increased energy consumption, therefore there is a need to manage network congestion control mechanisms.

In order to solve the problem of network performance degradation caused by the congestion of intermediate nodes, sharp declines in throughput, high packet loss rates, and other factors, a PI queue congestion algorithm, PID queue congestion algorithm, single neuron PID queue congestion algorithm and PNPID algorithm were simulated and compared. It can be concluded that the PNPID algorithm stabilized queue length near the expected value or lowered queue length at different transmission rates in most instances. The volatility of the queue length was also smaller, which showed the stability of the PNPID algorithm. At the same time, the throughput, packet loss rate and delay performance of the PNPID algorithm were improved, to some extent, compared to the other three algorithms, thus demonstrating the excellent performance of the PNPID algorithm.

However, the validity of the wireless sensor network congestion control method presented in this paper has only been verified in a network simulation environment. In the future, the performance of the algorithms in practical applications needs to be verified.

## Figures and Tables

**Table 1:** Physical meaning of each parameter in the PID queue congestion control model.

Parameters	The Meaning of the Parameters
p	packet dropping probability
$q_{0}$	expected queue length
q	actual queue length
$K_{p}$ $K_{i}$ $K_{d}$	the coefficients of proportion, integral and differentiation
e	input data variance, $e=q_{0}-q$

**Table 2:** The definition of each parameter in the WSN topological graph.

Parameters	Values
The node transmission range	250 m
The MAC layer protocol	IEEE 802.11
Routing protocol	AODV
Queue length of each sensor node	50 Packets
Expected queue length	20 Packets
Packet size	128 B
Sampling frequency	100 H_Z_
Simulation time	50 s
Simulation scenario	2980 m × 1278 m
Traffic	CBR

**Table 3:** Parameter settings of PI, PID, NPID, and PNPID.

Parameters	The Meaning of the Parameters
Traditional PI algorithm	Fixed parameter value $K_{p}$ = 0.0000475, $K_{i}$ = 0.0000174 [25]
Traditional PID algorithm	$K_{p}$ = 0.0000129, $K_{i}$ = 0.0000222, $K_{d}$ = 0.0000095 [26]
NPID algorithm	Neuronal gain is *K* = 0.12
PNPID algorithm	Neuronal gain is *K* = 0.12
